# Pyrosequencing the Canine Faecal Microbiota: Breadth and Depth of Biodiversity

**DOI:** 10.1371/journal.pone.0053115

**Published:** 2013-01-31

**Authors:** Daniel Hand, Corrin Wallis, Alison Colyer, Charles W. Penn

**Affiliations:** 1 School of Biosciences, University of Birmingham, Birmingham, United Kingdom; 2 WALTHAM® Centre for Pet Nutrition, Melton Mowbray, Leicestershire, United Kingdom; Baylor College of Medicine, United States of America

## Abstract

Mammalian intestinal microbiota remain poorly understood despite decades of interest and investigation by culture-based and other long-established methodologies. Using high-throughput sequencing technology we now report a detailed analysis of canine faecal microbiota. The study group of animals comprised eleven healthy adult miniature Schnauzer dogs of mixed sex and age, some closely related and all housed in kennel and pen accommodation on the same premises with similar feeding and exercise regimes. DNA was extracted from faecal specimens and subjected to PCR amplification of 16S rDNA, followed by sequencing of the 5′ region that included variable regions V1 and V2. Barcoded amplicons were sequenced by Roche-454 FLX high-throughput pyrosequencing. Sequences were assigned to taxa using the Ribosomal Database Project Bayesian classifier and revealed dominance of Fusobacterium and Bacteroidetes phyla. Differences between animals in the proportions of different taxa, among 10,000 reads per animal, were clear and not supportive of the concept of a “core microbiota”. Despite this variability in prominent genera, littermates were shown to have a more similar faecal microbial composition than unrelated dogs. Diversity of the microbiota was also assessed by assignment of sequence reads into operational taxonomic units (OTUs) at the level of 97% sequence identity. The OTU data were then subjected to rarefaction analysis and determination of Chao1 richness estimates. The data indicated that faecal microbiota comprised possibly as many as 500 to 1500 OTUs.

## Introduction

The intestinal microbiota can be defined as the total population of microbial species that inhabit the digestive tract. This community of organisms is increasingly recognised as a major contributor to the digestion and utilisation of foods in the gastrointestinal tract, and a key factor in nutrition, development, immune function and other aspects of host physiology that contribute to health and wellbeing [1], [2], [3], [4], [5], [6], [7].

The microbiota of the mammalian digestive tract are very numerous, diverse and complex in their composition, comprising at least hundreds, perhaps thousands of interdependent and/or competing species [8], [9], [10] that are generally incompletely characterised. The most diverse and abundant component of the digestive tract microbiota in monogastric mammals is the community associated with the contents of the large intestine. This community is usually dominated numerically by strictly anaerobic species, typically including members of the *Bacteroidetes* and *Clostridia* [11], [12]. These groups include numerous diverse and often uncultured genera and species. Other members of the Firmicutes (low G+C Gram-positives) including anaerobic cocci are also often abundant [12] whilst the Actinobacteria and Proteobacteria, the latter including many of the medically important pathogenic genera, are often less abundant but nevertheless significant members of the population.

Many species found in the gastrointestinal tract are largely uncharacterised, due to the difficulty of culturing them routinely in the laboratory [13], [14], although efforts are continuing to culture new species, with considerable success [15], [16]. Nevertheless the huge challenge posed by growth and characterisation of hundreds of often fastidious and highly diverse species has in practice made comprehensive and systematic culture-based analysis of mammalian gut microbiota an unattainable goal; furthermore it is a largely qualitative approach. In contrast, high throughput sequencing, for example by Roche-454 deep pyrosequencing of 16S rDNA amplicon pools now enables quantification of different microbial groups directly, based on the numbers of copies obtained of their signature sequences [17]. Typically, a million or more sequence reads can be obtained per instrument run, and sequence tags can be used to enable reads from numerous different samples to be binned and analysed thus enabling simultaneous processing of large numbers of samples.

A number of recently published studies now describe the exploitation of these technologies to analyse the gut microbiota of humans [18], [19] and other animal species such as non human primates [20], [21], mice [22], pigs [23], chickens [24], cats [25] and dogs [26], [27]. In humans, where interest in the microbiota is intense as its importance in dietary processing and health is increasingly recognised [19], the complexity of rigorous studies is compounded by human genetic diversity and the difficulty of defining and controlling dietary, behavioural, environmental and other variables in a study population. In contrast, studies in defined animal populations offer opportunities to control these variables. Dogs for example include inbred lines represented by different breeds. Animals housed and fed under similar and controlled regimes are not subject to many of the confounding variables of human study populations.

Knowledge of canine intestinal microbiota is much less complete than for humans. However, several studies investigating canine microbiota using culture-independent methods have now been published [27], [28], [29], [30], [31]. High throughput sequencing has been used to investigate the diversity of bacterial and fungal microbiota in canine faeces [25], to determine the effects of antibiotics on microbial diversity [26] and to study the effects of diet formulation [27] and dietary fibre on canine faecal bacterial phylogeny [32] and functional capacity [33]. However canine gut microbiota are yet to be systematically characterised at the species level, and knowledge based on culture methods [14], [29], [34], [35] is difficult to relate to the newer high-throughput culture-independent data. Thus there is no consensus view of the composition of ‘normal’ canine faecal microbiota, or the extent of its variation between individuals. We now describe the analysis of faecal microbiota in a group of miniature Schnauzer dogs housed on the same site and with known dietary intakes and genetic relatedness, and illustrate the potential for such an approach to generate fundamental new insights into canine gut microbiology.

## Results and Discussion

### Data acquisition and analysis

Pyrosequencing by Roche-454 GS FLX of faecal rDNA amplicons from 11 miniature Schnauzer dogs, including a replicate analysis of the MSs1 sample, yielded 247,501 reads representing 56.7 Mb of sequence. The average read length was 229 bp. After the raw sequence data were filtered for quality (see methods), which removed approximately 20% of reads (51,602 in total), sequences were binned by barcode to yield an average of 17,899± SD 1,434 reads per barcode.

**Figure 1:**
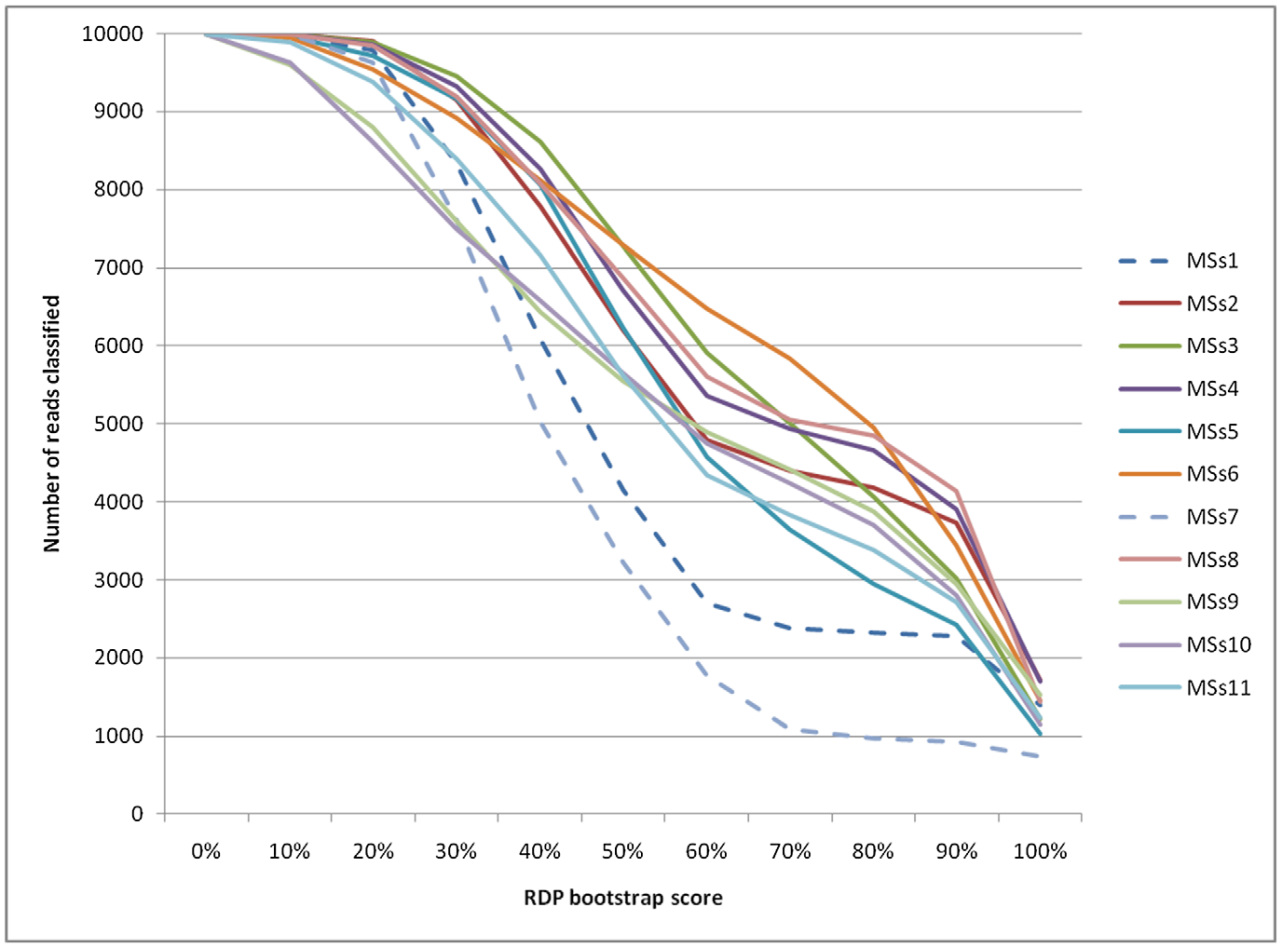
Effect of altering bootstrap score on assignment of sequence reads to genus level. The graph shows RDP bootstrap score (x axis) and number of reads that would be classified to the genus level (y axis) for each dog.

To make an initial assessment of the microbiota in each dog, the reads were analysed using the Bayesian classifier algorithm from the Ribosomal Database Project (RDP, http://rdp.cme.msu.edu/) [36]. This method was chosen to take advantage of the straightforward RDP pipeline, fast analysis and widely used, high quality sequence database. Identification of reads to the genus level with increasingly strict RDP classifier bootstrap values resulted in a large and uneven drop in assigned read numbers between the dogs (Fig. 1). Dogs MSs1 and MSs7 in particular were subject to disproportionate reductions in the proportion of reads that could be classified with increasing filtering stringency based on the bootstrap score. A possible explanation for this observation is given below in relation to the taxonomy of sequence reads from these dogs.

A bootstrap score of 50% has been recommended for genus level identification [37]. However, we decided to reduce this to 30% to maintain a more even representation of reads across the group of dogs, accepting the trade-off that a minority (estimated to be ∼15%, see methods) of classifications will be incorrect.

### Composition of microbial communities

First we combined RDP classified data from all eleven dogs to generate an overview of the microbiota composition, as represented by the read sequences. In the following discussion we assume that the presence of sequences reflects the presence of the corresponding organisms in the faecal biomass, with the caveat that DNA from dead bacteria or even potentially from ingested foods or other sources may persist and be detectable in the total faecal DNA fraction. There is very little literature available on which to estimate the stability of dietary DNA during passage through the digestive tract, but there is some evidence of persistence in the GI tract of recombinant (GM) DNA from dietary sources [38], [39].

We discovered that at the phylum level (see Table 1), the combined community was dominated by the Fusobacteria (39.17% of reads) followed by the Bacteroidetes (33.36%), Firmicutes (15.81%), Proteobacteria (11.31%), Actinobacteria (0.33%) and several other phyla at lower abundances. The clear abundance of sequences attributable to phylum Fusobacteria is somewhat unusual for a mammalian gut community [9], [40], [41], [42], and our observations echo findings of others in a study of dogs [32], although contrasting reports demonstrated greater dominance of Firmicutes, Actinobacteria and Bacteroidetes phyla [25], [27]. Thus there is little clear concensus on the predominant phyla present in dogs and the significance of an enriched Fusobacteria phylum in the dog microbiota is obscure. Notable in these data is the virtual absence of phylum Bacteroidetes in sample MSs1 and very low numbers in sample MSs7, although this phylum is well represented in all the other dogs; replication of this analysis confirmed the result so it does not appear to be due to any technical error. Furthermore, classification to phylum level of reads that could not be assigned to genera, as shown in Table 2, did not assign any of those reads to the Bacteroidetes phylum.

**Table 1:** Percentage of sequence reads assigned to each phylum using an RDP bootstrap score of 30%.

Phylum	MSs1	MSs1#2	MSs2	MSs3	MSs4	MSs5	MSs6	MSs7	MSs8	MSs9	MSs10	MSs11	Grand Total*	Percentage*
**Fusobacteria**	5600	5390	4334	3210	3489	3493	1593	4757	3616	1250	2099	3632	37073	39.17%
**Bacteroidetes**	1	1	2118	4073	4025	2154	5015	338	3711	3596	3708	2836	31575	33.36%
**Firmicutes**	1665	1814	1007	1278	855	1513	1638	2443	570	2376	828	792	14965	15.81%
**Proteobacteria**	1074	1045	1674	860	908	1981	658	26	1266	302	840	1112	10701	11.31%
**Actinobacteria**	4	2	18	35	33	30	17	49	20	66	19	21	312	0.33%
**Tenericutes**						13							13	0.01%
**Acidobacteria**									3				3	0.00%
**Deferribacteres**												3	3	0.00%
**Spirochaetes**												2	2	0.00%
**Chloroflexi**									1				1	0.00%
**Cyanobacteria**													0	0.00%
**Grand Total**	8344	8252	9151	9456	9323	9171	8921	7613	9187	7590	7494	8398	94648	100.00%

* Grand totals and percentages were calculated excluding the replicated data MSs1#2.

**Table 2:** Percentages of sequence reads, which were unassigned at the genus level using an RDP bootstrap score of 30%, that could be assigned by phylum.

Phylum	MSs1	MSs1#2	MSs2	MSs3	MSs4	MSs5	MSs6	MSs7	MSs8	MSs9	MSs10	MSs11	Average*
**Bacteroidetes**	0.00	0.02	0.00	0.03	1.19	0.25	3.02	0.00	0.04	8.14	10.18	4.91	2.52
**Firmicutes**	1.55	1.87	0.89	0.99	0.93	2.93	2.64	3.83	0.99	3.51	2.54	1.56	2.03
**Fusobacteria**	14.81	15.34	7.56	4.39	4.63	4.88	2.83	19.97	7.09	3.92	4.45	6.23	7.34
**Proteobacteria**	0.02	0.06	0.03	0.01	0.02	0.22	0.03	0.00	0.01	4.31	6.38	0.04	1.01
**Unclassified**	0.18	0.19	0.01	0.02	0.00	0.01	2.27	0.06	0.00	4.21	1.51	3.28	1.05
**Total**	16.56	17.48	8.49	5.44	6.77	8.29	10.79	23.86	8.13	24.09	25.06	16.02	13.95

* Averages were calculated excluding replicated data MSs1#2.

**Figure 2:**
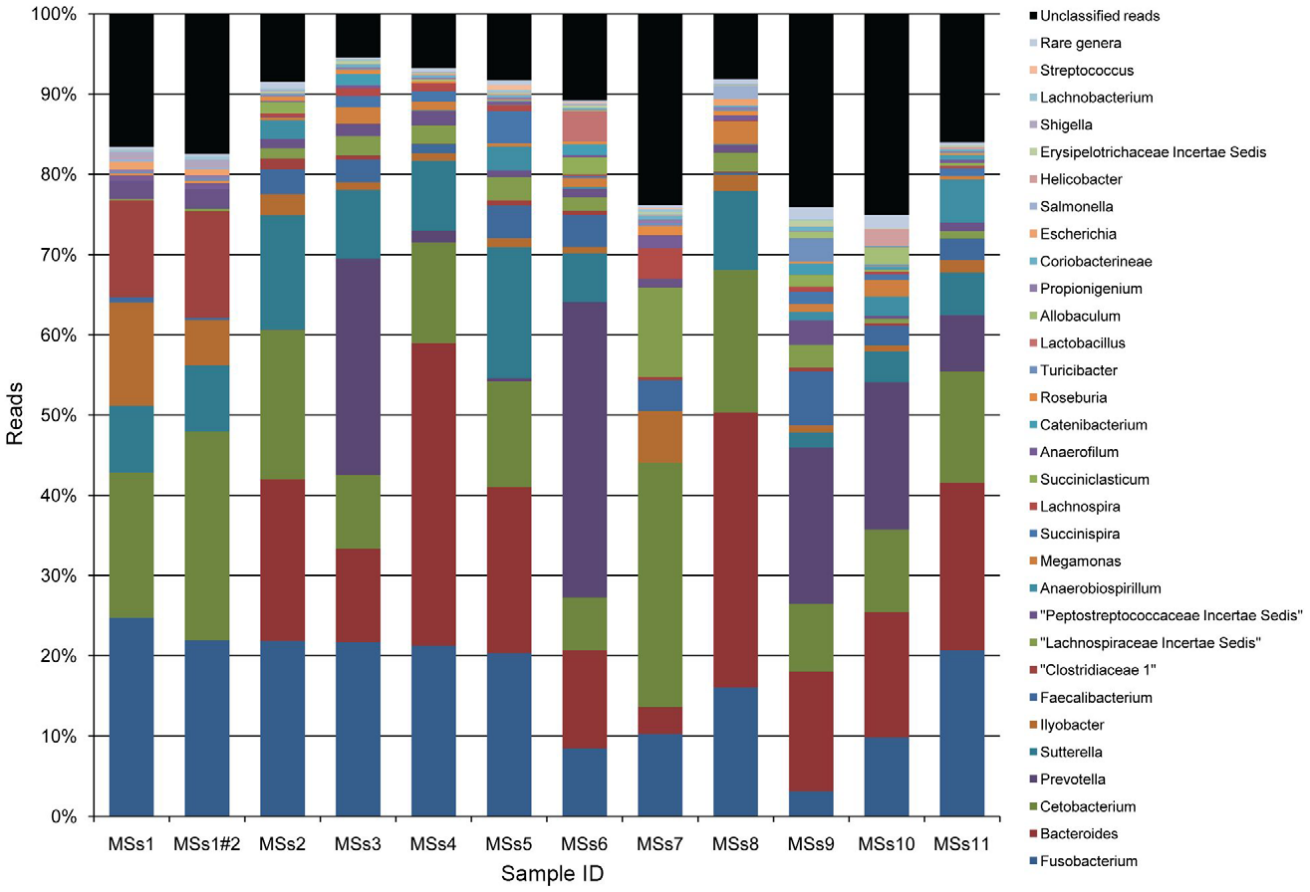
RDP classification of reads to the genus level. Sample number is shown on the x axis and percentage reads classified on the y axis. Genera with fewer than 100 reads in all samples were pooled and are shown as ‘rare genera’. MSs1#2 denotes replicated read data on the same DNA sample for dog MSs1. The complete data set is tabulated in table S1.

In addition to the phylum level inter-animal variation evident from Table 1, analyses at the genus level of the faecal microbiota from individual animals revealed considerable divergence in abundances of the major genus level taxonomic groups (Fig. 2). The percentage coefficient of variation (CV) for each genus was calculated on the log_10_ data, given as 100× SD/Mean. CVs ranged from 6 to 193% (with mean counts of 1347 and 1 respectively); the median CV was 44%. In most of the animals the five most abundant genera represented approximately 60–80% of the bacteria present. However even among the most abundant genera the data were highly variable between animals; only the prominent members of the Fusobacteria phylum (*Fusobacterium, Cetobacterium and Ilyobacterium*; CVs of 8.6% +/−0.27, 6.2% +/−0.2, and 16.5% +/−13.7 and mean counts of 1406, 1347 and 164 respectively) were among the most abundant groups in all animals. *Fusobacteria* have been shown to ferment carbohydrates and certain amino acids to produce butyrate, acetate and other volatile fatty acids [43]. *Cetobacteria* have been little-investigated but have been isolated from human faeces as well as fish and shown to ferment peptides and carbohydrates [44] and to produce vitamin B12 [45]. *Ilyobacter* has been characterised as a 3-hydroxybutyrate fermenting anaerobe [46], [47]. None of the other most abundant genera, *Bacteroides, Prevotella* and *Sutterella*, were highly abundant in every one of the dogs tested. Notably, members of the little-investigated β-Proteobacterial genus *Sutterella* accounted for a large majority of Proteobacteria phylum members. The observed divergence between animals does not support the concept of a ‘core microbiota’ at the genus level in which the major constituents of the community will be more or less universally present at comparable levels in different individual hosts. This finding is in agreement with studies in humans [48], [49]. A study of microbiota in monozygotic and dizygotic twins [19] indicated that no species-level phylotype at an abundance ≥∼0.5% of the total number of phylotypes in all of the samples from a total of 154 human individuals was universally present. Faeces collected from Koreans was found to contain even lower levels of shared microbiota, with only 0.005% of species-level phylotypes represented in at least 75% of individuals [50]. Other studies of the human intestinal microbiota have reported the levels of shared microbiota to be slightly higher with 2.1% of species-level phylotypes being present in more than 50% of the samples [51].

Among prominent genera identified in this study, the combined *Prevotella* and *Bacteroides* (phylum Bacteroidetes) abundances tended to be inversely related to phylum Fusobacteria in abundance. We hypothesise that this distribution may relate to ‘competition’ for the same niche by these groups of bacteria. This type of relationship has also been observed in human studies where the combined contributions of Firmicutes and Bacteroidetes have been shown to remain largely constant over time and between individuals [50]. A hypothesis regarding this type of relationship is that the gastrointestinal gene pool remains largely constant throughout life but the microbes themselves are continuously replaced in response to environmental change.

As mentioned above, samples from dogs MSs1 and MSs7 were subject to a disproportionate reduction in the number of reads that could be classified to genus level, as the classifier bootstrap score was increased above 30%. In assignment to phyla of the sequences that could not be classified to genus level using a 30% RDP bootstrap score, faeces from dogs MSs1 and MSs7 yielded more Fusobacteria reads (15–20%) than the other dogs (3–8% of reads) (see Table 2). The literature on isolation or detection of *Fusobacteria* (with the exception of *Fusobacterium prausnitzii*, reassigned to the *Firmicutes*, family *Ruminococcaceae* as *Faecalibacterium prausnitzii* in 2002 [52]) from intestinal contents or faeces is sparse [53], [54], [55], and indicates that these organisms are not often prominent in the human gut microbiota. It has been suggested that their association with the mucosa may make isolation difficult [56]. The low numbers isolated and characterised in the past may also explain the paucity of representative sequences in the RDP database, hence limiting ability to classify these sequences to genus level.

### Extent of coverage of microbiota diversity

**Figure 3:**
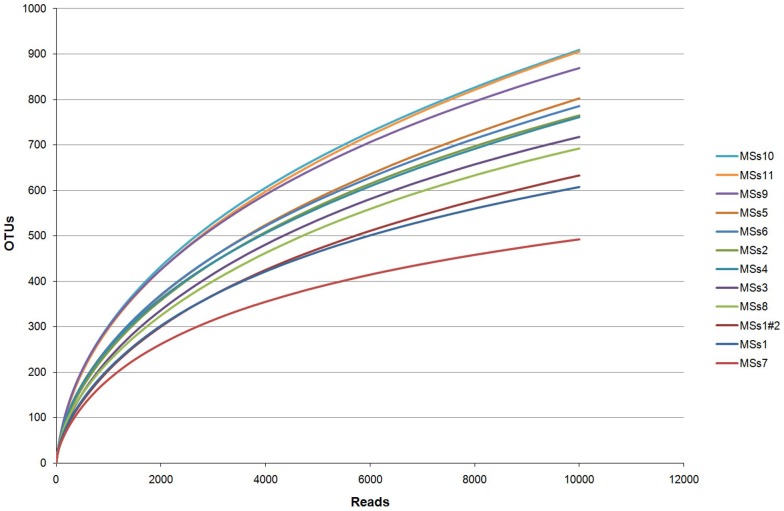
Rarefaction curves for each study animal. Number of reads is shown on the x axis and number of OTUs at 97% sequence identity on the y axis. Also shown are the technical replicates of the analysis of DNA from MSs1.

The overall extent of diversity of the digestive tract microbiota is an important question in terms of understanding its complexity and biological role. The identification of sequence reads by matching them to known sequences in the RDP database clearly has limitations in determining overall diversity, since sequences that do not match well to known organisms have been discarded from the analysis above. This is particularly relevant for identification of microbes from less well studied hosts. We therefore determined the diversity of sequences based on the allocation of reads to ‘operational taxonomic units’ or OTUs, independently from any known sequence homologies, using RDP infernal aligner and complete linkage clustering tools [57]. The OTUs represent notional taxa, and at a 97% sequence identity level (i.e. 3% sequence difference from others), reads within an OTU are likely sampled from the same species and individual OTUs approximate to different species. Data showing diversity in an ecological context, where a habitat may not have been exhaustively sampled, can conveniently be analysed by mathematically modelling the occurrence of ‘new’ and repeat sequences in the sample to predict the total diversity in the system. Such analyses can be based on rarefaction curves, shown in Fig. 3, where it can be seen that by these analyses there appear to be significant additional numbers of OTUs yet to be discovered.

**Figure 4:**
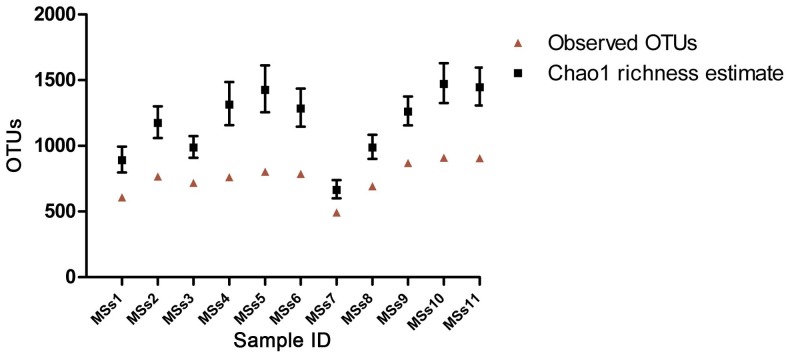
Observed OTUs calculated using the RDP Infernal Aligner and Complete Linkage Clustering tools, and Chao1 richness estimates calculated using Mothur, based on the 10,000 reads analysed from each animal. Red triangles: observed OTUs; black squares: Chao1 richness estimates; bars represent upper and lower 95% confidence intervals.

It is also possible to predict total numbers of OTUs present in such samples by Chao1 richness estimate as shown in Fig. 4. As might be expected from the smaller proportion of the total microbiota taken up by the five most abundant genera in this animal, MSs9 shows a larger estimate of total microbiota diversity than, for example, MSs1. From the fact that the confidence intervals from these two ChaoI estimates do not overlap we can infer that these dogs differ significantly in their estimated species diversity. Overall it appears that in terms of OTUs, faecal microbiota diversity is likely to range from approximately 500 to 1500 OTUs per animal. However these estimates should be viewed with caution, as errors in Roche-454 sequencing at homopolymeric runs may contribute to an overestimate of diversity in the determination of OTU numbers [58]. Furthermore erroneous sequences arising from, for example, chimeric sequence formation resulting from DNA-DNA hybridisation between unrelated sequences during PCR amplification were not excluded in this study. Since completing this analysis we have assessed chimera occurrence in a very similar data set using the Perseus algorithm [59] and numbers of sequences discarded as chimeras were small, not exceeding 1% of the total, hence there may be small numbers of chimeric sequences present in our data set. Despite these reservations it is clear that there is substantial variation between animals in the diversity of the faecal microbiota.

### Genetic relatedness between animals and variation in microbiota

There were several closely related animals in this study cohort (see Table 3). Dogs MSs3, MSs4, MSs5, MSs6 and MSs10 were siblings, the first four being littermates. MSs5 was the mother of MSs7 and MSs8, while MSs8 was the mother of MSs9 and MSs11. MSE was the father of six of the study dogs. Furthermore there were some dietary differences (see Table 3), but 8 of the 11 dogs were fed Pedigree Adult Small Bite (SB). Although this study was not designed to be statistically powered for assessment of genetic or dietary influence on the microbiota, there were some indications of their effects.

**Table 3:** Details of miniature Schnauzer dogs used in the study, showing genetic relatedness.

Dog ID*	Sex^†^	Mother ID	Father ID	DOB	Age (yrs: mo)	Diet
MSs1	MN	MSA	MSC	01/06/1995	11:4	PEDIGREE STANDARD WET
MSs2	FN	MSA	MSD	16/09/1995	11:1	LOW FAT DRY+WET (VET DIET)
MSs3	MN	MSB	MSE	25/11/2000	5:11	PEDIGREE ADULT DRY SB
MSs4	FN	MSB	MSE	25/11/2000	5:11	PEDIGREE ADULT DRY SB
MSs5	FN	MSB	MSE	25/11/2000	5:11	TRIAL DIET BS0670
MSs6	FN	MSB	MSE	25/11/2000	5:11	PEDIGREE ADULT DRY SB
MSs7	MN	MSs5	MSF	18/04/2002	4:6	PEDIGREE ADULT DRY SB
MSs8	FE	MSs5	MSF	18/04/2002	4:6	PEDIGREE ADULT DRY SB
MSs9	FN	MSs8	MSE	23/06/2004	2:4	PEDIGREE ADULT DRY SB
MSs10	MN	MSB	MSE	04/07/2004	2:4	PEDIGREE ADULT DRY SB
MSs11	FE	MSs8	MSG	12/08/2005	1:2	PEDIGREE ADULT DRY SB

* MSs denotes miniature Schnauzers sampled during the study. ^†^Sex is shown as MN/FN/FE indicating male (M) or female (F) neutered (N) or entire (E) at the time of the study.

**Figure 5:**
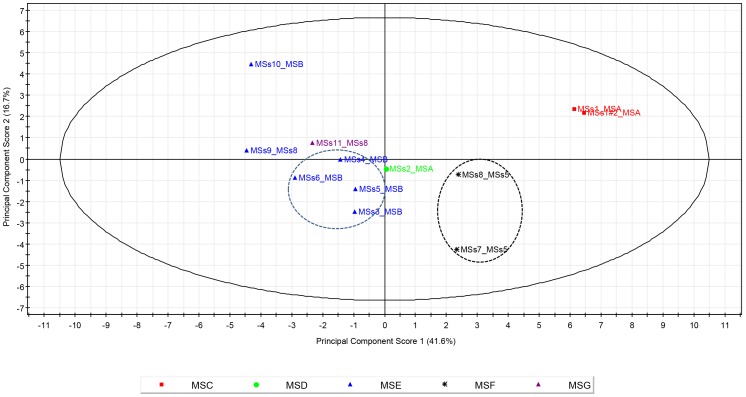
Principal component analysis on the log_10_ (count+1) of each of the most abundant genera (present at >0.1%) identified for each animal. Each point is labelled with the dog ID followed by the mother ID and is coloured according to the father ID; red (MSC), green (MSD), blue (MSE), black (MSF) and purple (MSG). Dotted circles identify littermates.

Principal component analysis (PCA) on the log_10_ (counts +1) of the most abundant genera in the microbiota of individual dogs revealed clustering of samples obtained from genetically related animals (see figure 5). There was an apparent grouping of littermates MSs3, MSs4, MSs5 and MSs6, despite MSs5 receiving a different diet from the other three littermates (see Table 3). The PCA loadings plot (not shown) indicated that this clustering was predominantly due to these dogs having higher levels of *Lactobacillus, Streptococcus, Lachnospiraceae, Faecalibacterium, Bacteroides, Lachnospira, Coriobacterineae and Erysipelotrichaceae*. In addition, dogs MSs9, MSs10 and MSs11 were more correlated with the group of four littermates than the other dogs. This may be due to the fact that they were also closely related; MSs10 was a sibling of the littermates, MSs9 shared the same father as MSs10 and the littermates and MSs9 and MSs11 shared the same mother. Dogs MSs7 and MSs8 were also littermates and formed another cluster predominantly due to higher levels of *Roseburia, Lachnobacterium, Propionigenium, Anaerofilum, Peptostreptococcaceae and Cetobacterium*. MSs1 and MSs2 shared the same mother but also received different diets to the majority of other dogs which might also explain their lower level of correlation with other animals. MSs1 received a standard Pedigree wet diet and MSs2 was on a veterinary diet whereas all the other dogs received Pedigree adult small bite (SB) with the exception of MSs5 which was receiving a trial diet at the time of faeces collection.

The findings are in contrast to other canine studies where no discernable effect of littermates was noted in gut microbial composition in a cohort of six dogs comprising three pairs of littermates [32]. Our data are only indicative and a larger study including more littermates is required to confirm these preliminary findings. However, studies in mice and humans have shown that genetically related individuals have a more similar gut microbial composition than unrelated individuals [60], [61]. In contrast, a study of gut microbiota in pairs of twins and their mothers found no significant difference in degrees of similarity in gut populations [19]. These contradictory findings suggest that other factors as well as host genotype are likely to contribute to the composition of the intestinal microbial community.

A control was included in the analysis whereby DNA from the same sample (MSs1) was analysed (amplified and sequenced) in duplicate. The distribution of genera within these replicate data sets was more similar than between any two individual animals (figure 5), indicating that the variations seen between animals are not solely due to technical error in the analysis. Clustering of log_10_ (count +1) of genera showed that the repetitions for MSs1 are correlated to 0.99.

### Summary and biological significance

The data described comprise one of the first studies to date of the faecal microbiota in a number of closely related dogs, in terms of both breed and family relationships. Considerable differences between individuals were observed, especially in quantitative terms, between the major groups of bacteria detected, albeit a broad similarity is also perceptible in the dominance of anaerobic organisms in the Fusobacteria and Bacteroidetes phyla. This study also shows that genetically related dogs have a more similar faecal microbial composition than unrelated dogs. Taken together, these finding suggest that genetics along with other factors such as diet and age are likely to contribute to shaping the composition of the canine intestinal microbial community. We emphasise however that due to the lack of prior information about biological variation in this system, we have not achieved the necessary statistical power in this study to claim statistical significance in the majority of our observations.

## Materials and Methods

### Animals and faeces collection

The eleven study animals were healthy adult Miniature Schnauzer dogs housed at the WALTHAM® Centre for Pet Nutrition, and many of the group were closely related genetically. Details are given in Table 1. The dogs were pair-housed in high quality kennel accommodation which exceeded both Home Office and European regulatory requirements. All dogs had constant inside and outside access, including access to paddocks throughout the day, and received similar levels of on- and off-lead exercise. Dogs had varying degrees of cross-contact from sharing pens and during exercise.

Dogs received a variety of diets as detailed in Table 3. One faecal sample was collected per dog during daily exercise and a cross-section of stool to include both surface and internal content (approx 2 g) was frozen on dry ice no more than 15 minutes following defaecation. The frozen samples were stored at −80°C for between one and two weeks before DNA extraction.

### Faecal DNA extraction

Faecal DNA was extracted from faecal samples using a QIAamp DNA Stool Mini kit (Qiagen). A subsample of 190–220 mg of frozen faeces was processed following the ‘Protocol for Isolation of DNA from Stool for Pathogen Detection’ detailed in the manufacturer's instructions, using a lysis temperature of 95°C.

Extracted DNA was eluted from the spin columns in 200 µl of Qiagen AE buffer (10 mM Tris-Cl and 0.5 mM pH 9.0 EDTA). Extracted DNA was then quantified and checked for purity (based on UV absorption spectrum and 260∶280 nm and 260∶230 nm absorption ratios) on a ND1000 spectrometer (Nanodrop Technologies Inc) and samples with poor yields (<∼15 ng/μL) and/or highly aberrant absorption ratios were were re-extracted.

### Amplification by PCR of 16S rDNA

A barcoded 16 S rDNA tag approach was used to amplify a ∼500 bp region (bases 28–514, excluding primer annealing regions in the *E. coli* sequence) which includes the V1, V2 and V3 regions of the 16 S rDNA sequence, although only the 5′∼230 bases including barcode and primer were sequenced which included variable regions V1 and V2. The primer sequences were as follows: forward primer 5′-GCCTCCCTCGCGCCATCAG[N8]AGAGTTTGATYMTGGCTCAG-3′ and reverse primer, 5′-GCCTTGCCAGCCCGCTCAGTIACCGIIICTICTGGCAC-3′. The forward primer comprised, from the 5′ end, the 454 sequencing adapter A, a sample barcode octamer (denoted by N8) and rDNA-specific sequence 27f-YM [62]. The reverse primer comprised from the 5′ end, 454 sequencing adapter B and rDNA-specific sequence I533r [63]. Individual 50 µl PCRs were set up as follows; 25 µl Extensor ready mix (Thermo Scientific), 3 µl of each primer (10 pmol/µl), 1.5 µl Nuclease-Free Water (Promega) and 17.5 µl (35 ng) faecal DNA. Amplification was for 30 cycles with the following conditions: 94°C for 3 min:00s (3:00) followed by 10 cycles of 94°C for 0:45, 55°C for 0:30, 72°C for 1:00, then 19 cycles of 94°C for 0:45, 55°C for 0:30, 72°C for 1:30, and a final cycle of 94°C for 0:45, 55°C for 0:30 and 72°C for 7:00. Amplicon abundance and size were checked using agarose gel electrophoresis and were then purified using a QIAquick PCR Purification Kit (Qiagen).

### Roche-454 sequencing

Purified PCR amplicons from different dogs were pooled on an equimolar basis based on ND1000 spectrometer readings. This pool was then sequenced from a primer annealing to adapter A on a 454 GS FLX sequencer (Roche) using FLX chemistry and picotitre plates following the manufacturer's protocols.

### Data analysis

Using local databases and code written in Python, the raw sequence read data were initially filtered to remove sequences below 150 nt, those containing one or more ambiguous bases and those with a mismatch against the 27f-YM primer sequence. Sequences were then binned by barcode sequence and each bin was randomly resampled using the Random module in Python, based on the Mersenne Twister algorithm [64]. This allowed standardisation of sequence read number to 10,000 reads per dog, the highest ‘round number’ that would not exclude any of the dogs, to reduce bias in the subsequent comparative data analysis between dogs. This was deemed necessary because it is well recognised that estimates of species richness are dependent on sample size [65], [66], and a recent report indicates that equalisation of sample sizes is crucial in comparisons between samples [67].

Taxonomic assignment of reads was done using a downloaded copy of the 2.0 version of the Bayesian classifier algorithm from the Ribosomal Database Project (RDP, http://rdp.cme.msu.edu/) [36]. Classification at the phylum and genus levels was done using bootstrap scores ranging from 0% to 100%. A value of 30% was selected because although a significant number of genus identifications are likely to be incorrect, a substantially greater number of correctly identified genera are then included in the output data; the value of a high level of sampling has been emphasised in a recent review [67]. The number of incorrectly classified reads at a bootstrap value of 30% was estimated be approximately 15% based on *in silico* modelling of the classifications of a set of ∼250 sequences of known taxa trimmed to 250 bp spanning regions V1 and V2 (data not shown). We believe that on balance the use of this bootstrap value increases the validity of the determination of proportions of different genera present.

Operational taxonomic units (OTUs) were determined for the total of 120,000 reads combined from the 10,000 resampled reads per dog, using the RDP infernal aligner and Complete Linkage Clustering tools [57]. Rarefaction curves and Chao1 richness estimates [68], [69] were calculated for each dog using Mothur version 1.8.1 [70].

### Statistical analysis

Principal component analysis (PCA) was performed on the log_10_ (counts +1 to allow for zeros) to determine if variability of the most abundant genera (>0.1% of total reads) was associated with gender, mother, father, sibling group or diet. Genera present at <0.1% in all 12 samples were classified in a single group of “rare” taxa, and reads that were unclassified, using the predefined criteria, formed another group. All analyses were performed in SIMCA-P version 10 (Umetrics).

## Supporting Information

Table S1 Text file of complete tab delimited data output from RDP classification of reads to the genus level used for generation of Fig. 2.
(7Z) Click here for additional data file.
